# Correlation of OXA-1 and TEM-1 genes with antibiotic resistance to piperacillin/tazobactam in ESBL-producing Enterobacterales: insights from a multi-center analysis

**DOI:** 10.3389/fcimb.2025.1694724

**Published:** 2026-01-12

**Authors:** Edwin Kamau, Brendan M. Wong, John L. MacArthur, Jamie L. Dombach

**Affiliations:** 1 Department of Pathology and Area Laboratory Services, Tripler Army Medical Center, Honolulu, HI, United States; 2 Adult Intensive Care Unit, Tripler Army Medical Center, Honolulu, HI, United States; 3 Department of Clinical Investigation, Tripler Army Medical Center, Honolulu, HI, United States

**Keywords:** Extended-spectrum β-lactamase (ESBL), OXA-1 β-lactamase, TEM-1 β-lactamase, CTX-M-15, Piperacillin/tazobactam resistance, *Escherichia coli*, *Klebsiella pneumoniae*, antimicrobial resistance

## Abstract

**Background:**

The emergence of extended-spectrum β-lactamase (ESBL)-producing *Escherichia coli* and *Klebsiella pneumoniae* presents significant challenges in treating infections caused by these pathogens. This multi-center retrospective study investigated the prevalence of OXA-1 and TEM-1 genes in ESBL-producing *E. coli* and *K. pneumoniae*, along with their association with piperacillin/tazobactam susceptibility and additional antimicrobial resistance genes.

**Methods:**

Clinical isolates were collected from three institutions as part of routine patient care: Tripler Army Medical Center (TAMC) in Hawaii, Madigan Army Medical Center (MAMC) in Washington, and Brooke Army Medical Center (BAMC) in Southern Texas. A total of 416 isolates were analyzed through genome sequencing and CLSI-guided susceptibility testing.

**Results:**

OXA-1 and TEM-1 β-lactamase enzymes were present in 20.9% (73/349) and 38.7% (135/349) of the *E. coli* isolates, respectively. Relative risk analysis of non-susceptibility to piperacillin/tazobactam across isolates from the three study sites revealed a highly significant association for OXA-1 (*P* < 0.001), whereas no significant associations were observed for TEM-1 (*P* = 0.424) or the combination of OXA-1 and TEM-1 (*P* = 0.082). When analyzed by institution, the relative risk of non-susceptibility to piperacillin/tazobactam remained highly significant for OXA-1 at TAMC and MAMC (*P* < 0.001 for both) but was not significant at BAMC (*P* = 0.21). OXA-1 and TEM-1-positive variants showed a significant association with genes conferring resistance to other antibiotics.

**Conclusions:**

The OXA-1 gene plays a key role in resistance to piperacillin/tazobactam in ESBL-producing organisms, with geographic differences in non-susceptibility observed. Genetic profiling and localized data are crucial for optimizing antibiotic therapy and improving treatment outcomes.

## Introduction

Extended-spectrum β-lactamase (ESBL)-producing Gram-negative pathogens have emerged as a significant public health threat since their discovery in the early 1980s. These pathogens have developed resistance to expanded-spectrum β-lactam antibiotics, posing challenges in both hospital-associated (HA) and community-acquired (CA) infections (Drawz and Bonomo, 2010; Tufa et al., 2020; Castanheira et al., 2021). The global spread of ESBL-producing Enterobacterales has necessitated the exploration of alternative treatment options to combat these resistant strains (Castanheira et al., 2021).

Piperacillin/tazobactam has been proposed as a potential carbapenem-sparing alternative for managing infections caused by ESBL-producing *Escherichia coli* and other Enterobacterales. However, clinical studies on the efficacy of penicillin/inhibitor combinations against ESBL-producers have yielded contradictory results (Tamma and Rodriguez-Bano, 2017; Harris et al., 2018; Henderson et al., 2021; Hoashi et al., 2022; Onorato et al., 2025). The MERINO trial, a sentinel study investigating bacteremia due to ESBL strains, reported a 30-day mortality rate of 12.3% for patients treated with piperacillin/tazobactam compared to 3.7% for those treated with meropenem (*P* = 0.002) (Tamma and Rodriguez-Bano, 2017). *Post-hoc* analysis of the MERINO trial suggested that narrow-spectrum β-lactamases played a significant role in the poor performance of piperacillin/tazobactam compared to meropenem (Henderson et al., 2021). Importantly, the clinical outcome for patients receiving piperacillin/tazobactam may depend, at least in part, on the minimum inhibitory concentration (MIC) for this antimicrobial (Hoashi et al., 2022).

Since the MERINO trial, studies have demonstrated that several factors contribute to the variable resistance observed in ESBL-producers when treated with penicillin/inhibitor combinations. These factors include the production of multiple β-lactamases, such as OXA-1 and TEM-1, which are poorly inhibited by these combinations (Livermore et al., 2019; Walkty et al., 2022; Tamma et al., 2024). Additionally, hyperproduction of target β-lactamases and bacterial impermeability further complicate treatment outcomes. A study conducted by Livermore et al. assessing ESBL-producing isolates from the United Kingdom found that the presence of OXA-1 but not TEM-1 was strongly associated with reduced susceptibility to piperacillin/tazobactam and amoxicillin/clavulanate (Livermore et al., 2019), highlighting the enzyme’s role in resistance mechanisms. Similar findings were demonstrated in ESBL-producing isolates from Canada (Walkty et al., 2022). A study conducted by Rodríguez-Villodres demonstrated that piperacillin/tazobactam was less effective against ESBL *E. coli* carrying *bla*_TEM_ due to increase in copy numbers and transcription levels of the gene, and patients were at high risk of therapeutic failure (Delgado-Valverde et al., 2016). These findings underscore the importance of considering the presence of OXA-1 and TEM-1 when evaluating the efficacy of penicillin/inhibitor combinations against ESBL-producers.

As in most settings, piperacillin/tazobactam is one of the most widely prescribed empiric treatment options in military treatment facilities (MTFs) within the Defense Health Agency (DHA). In this study, we performed a multi-site retrospective study to investigate the prevalence of OXA-1 and TEM-1 in ESBL-producing *E. coli* and *Klebsiella pneumoniae* isolates in our patient populations. We explored the association between the minimal inhibitory concentrations (MICs) of piperacillin/tazobactam and the presence of OXA-1 and/or TEM-1 carrying ESBL isolates.

## Results

A total of 416 ESBL-producing organisms, 349 *E. coli* and 67 K*. pneumoniae*, were analyzed in this study. There were 106 isolates from Tripler Army Medical Center (TAMC) in Hawaii, 94 isolates from Madigan Army Medical Center (MAMC) in Washington, and 216 isolates from Brooke Army Medical Center (BAMC) in Southern Texas, of which 93, 85, and 171 were *E. coli* (**Supplementary Table 1**). BAMC had the highest proportion of ST131 isolates, while MAMC had the lowest and ST1193 and ST998 were most prevalent in TAMC and lowest in BAMC, however seven of the eight ST648 isolates were found in BAMC (**Table 1**). Most isolates harbored a CTX-M ESBL enzyme, predominantly the CTX-M-15 β-lactamase, which was present in 50.1% (175/349) of the isolates, with 103/175 being ST131. The narrow-spectrum OXA-1 and TEM-1 β-lactamase enzymes were present in 20.9% and 38.7% of the 349 *E. coli* isolates, respectively. Of the 175 CTX-M-15 ESBL variants, 41.7% were OXA-1 positive, and 34.3% were TEM-1 positive. Of the CTX-M-15/ST131 isolates, 54.4% (56/103) were OXA-1 positive, and 36.9% (38/103) were TEM-1 positive; OXA-1 was only detected in the CTX-M-15 ESBL type variant. Other notable β-lactamase variants identified included CTX-M-27 (n=98, 28.1%), CTX-M-14 (n=36, 10.3%), CTX-M-55 (n=24, 6.9%), and CTX-M-3 (n=8, 2.3%). For *K. pneumoniae*, 92.5% had known STs, with the two predominant STs being ST307 and ST45.

**Table 1:** ESBL isolates from each of the sample locations and the breakdown of the most common sequence types.

*E. coli*	TAMC	MAMC	BAMC
ST10	1	2	7
ST38	5	5	6
ST69	2	2	6
ST131	45	40	98
ST636	1	4	4
ST648	0	1	7
ST998	11	2	1
ST1193	11	7	8
Other	17	22	34
Total	93	85	171
*K. pneumoniae*	TAMC	MAMC	BAMC
ST45	1	3	4
ST307	2	1	6
Other	10	5	35
Total	13	9	45

The highest proportion of isolates carrying CTX-M-15 was found in BAMC (59.1% [101/171]), while the lowest was in TAMC (36.6% [34/93]). We evaluated whether there was a significant difference in the proportion of CTX-M-15 variants between institutions and determined there was a significant difference (χ^2^ = 12.63; *P* = 0.0018). Pairwise comparison for the proportion test revealed no difference in the CTX-M-15 variant between TAMC and MAMC (*P* = 0.156), and MAMC and BAMC (*P* = 0.069). However, there was a difference between TAMC and BAMC (*P* < 0.001), revealing that the significant difference was primarily between TAMC and BAMC. For the CTX-M-27 ESBL variant type, TAMC had the highest proportion of isolates (38.7% [34/93]), whereas the lowest proportion was in BAMC (24.0% [41/171]). Analysis revealed a statistically significant association between the institutions and the distribution of the CTX-M-27 variant (*P* = 0.029), specifically between TAMC and MAMC (*P* = 0.045), and TAMC and BAMC (*P* = 0.012), but not between MAMC and BAMC (*P* = 0.898). This indicated that the significant differences were primarily between TAMC and both MAMC and BAMC. For the CTX-M-14 ESBL variant type, TAMC (15.0% [14/93]) had the highest proportion, while the lowest was in BAMC (5.8% [10/171]), suggesting that the distribution of this variant was significantly associated with the institution (χ^2^ = 7.27; *P* = 0.026). Pairwise comparison for the proportion test revealed a significant difference in the proportion of the CTX-M-14 variant between TAMC and BAMC (*P* = 0.013), and MAMC and BAMC (*P* = 0.026), but not between TAMC and MAMC (*P* = 0.860).

**Figure 1:**
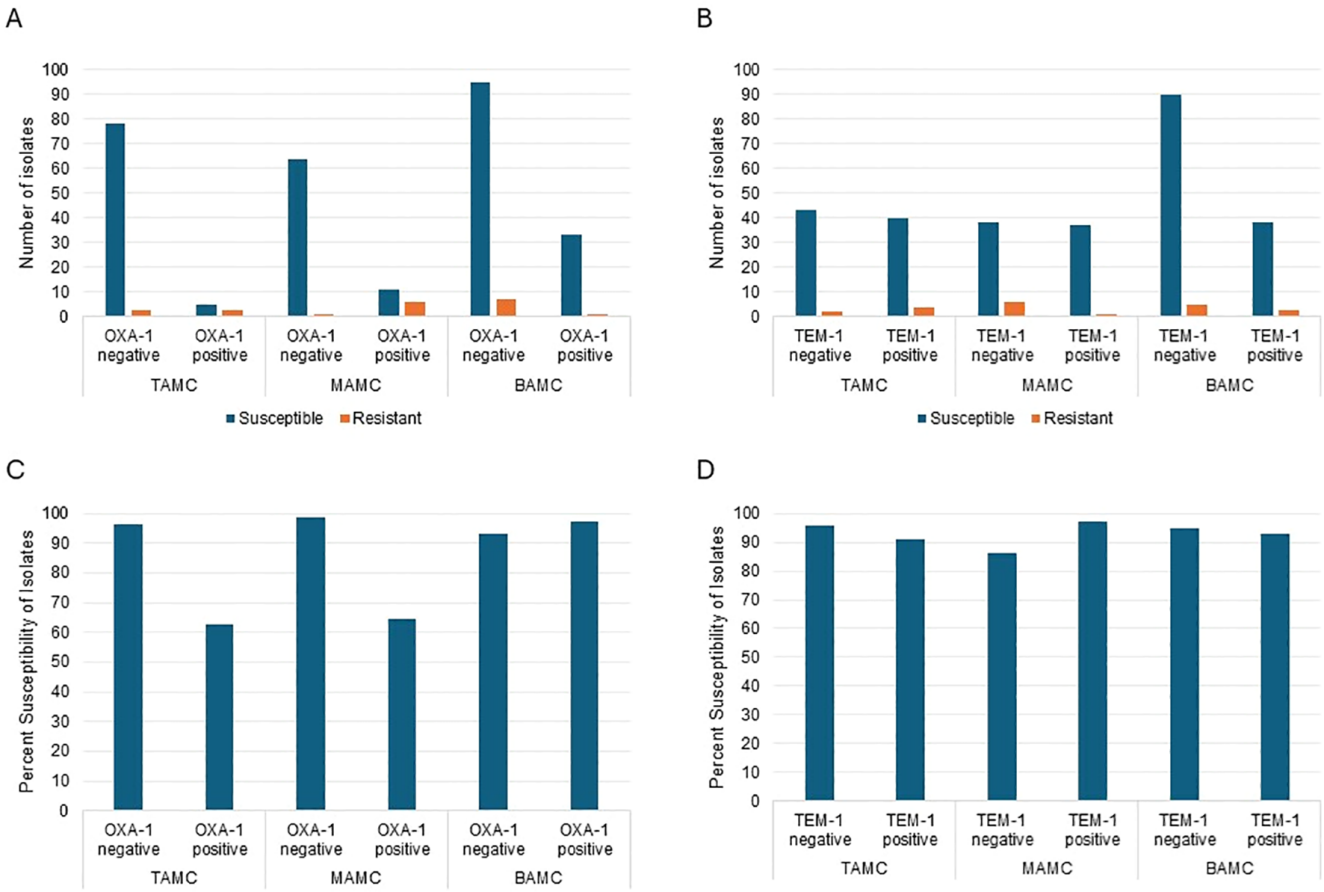
Analysis of *E. coli* isolates carrying OXA-1 or TEM-1 at each of the study sites **(A, B)** and the percent of isolates susceptible to piperacillin/tazobactam **(C, D)**.

Using the current Clinical and Laboratory Standards Institute (CLSI) susceptibility breakpoint of ≤8 mg/L for Enterobacterales, we examined the association between the presence of the OXA-1 or TEM-1 genes and susceptibility to piperacillin/tazobactam (CLSI, 2024). MIC data were available for 88.8% (310/349) of the isolates, of which 92.3% (286/310) were susceptible to piperacillin/tazobactam. Specifically, 95.6% (237/248) of OXA-1 negative isolates were susceptible to piperacillin/tazobactam, compared to 83.9% (52/62) of OXA-1 positive isolates. BAMC had the highest number of *E. coli* isolates that were OXA-1 positive, and TAMC had the fewest (**Figure 1A**). On the contrary, isolates positive or negative for TEM-1 were much more consistent at all study sites, except BAMC had an increased amount of TEM-1 negative isolates (**Figure 1B**). Notably, OXA-1 negative isolates exhibited significantly higher susceptibility in TAMC and compared to BAMC, where OXA-1 negative isolates had lower susceptibility (93.1%) than OXA-1 positive isolates (97.1%) (**Figure 1C**). For all three study sites the presence of TEM-1 had little effect on piperacillin/tazobactam susceptibility (**Figure 1D**).

Next, we evaluated the association between the presence of the OXA-1 gene and susceptibility to piperacillin/tazobactam in all ESBL-producing isolates in the three study sites and found a significant association between the presence of OXA-1 and non-susceptibility to piperacillin/tazobactam (χ^2^ = 6.99; *P* = 0.0082). Notably, all OXA-1 isolates were CTX-M-15, representing 41.7% (73/175) of this variant type. We found a significant difference in susceptibility to piperacillin/tazobactam between OXA-1 positive and OXA-1 negative *E. coli* bacteria *(P=* 0.0074). We analyzed the data by institution for the association between the presence of the OXA-1 gene and susceptibility to piperacillin/tazobactam across the three institutions (**Table 2**). Data from TAMC and MAMC demonstrated a statistically significant association, whereas no such association was observed at BAMC.

**Table 2:** Association of the presence of OXA-1 and susceptibility to piperacillin/tazobactam across the three institutions in this study.

Institution	Category	Chi-square	P	Z-statistic	P-value (proportion)
TAMC	Susceptible	6.72	**0.0095**	2.55	**0.0107**
MAMC	Susceptible	14.29	**0.0002**	3.78	**0.0002**
BAMC	Susceptible	0.92	0.337	0.96	0.337

Statistically significant values shown in bold.

We next assessed the association between the TEM-1 β-lactamase enzyme and susceptibility to piperacillin/tazobactam. Among all ESBL-producing isolates, chi-square tests for independence and proportion tests indicated no statistically significant association between the presence of the TEM-1 gene and susceptibility to piperacillin/tazobactam (data not shown). Among the CTX-M-15 isolates, 34.3% (60/175) were TEM-1 positive. Of these, 150 isolates (55 TEM-1 positive and 95 TEM-1 negative) had minimum inhibitory concentration (MIC) data available for piperacillin/tazobactam. There was no significant association between the presence of the TEM-1 gene and susceptibility to piperacillin/tazobactam.

We conducted a relative risk analysis of non-susceptibility to piperacillin/tazobactam across isolates from the three study sites. The analysis revealed a highly significant association for OXA-1 (*P* < 0.001), whereas no significant associations were observed for TEM-1 (*P* = 0.424) or the combination of OXA-1 and TEM-1 (*P* = 0.082) (**Table 3**). When analyzed by institution, the relative risk of non-susceptibility to piperacillin/tazobactam remained highly significant for OXA-1 at TAMC and MAMC (*P* < 0.001 for both) but was not significant at BAMC (*P* = 0.21). These findings were consistent with the results of chi-square tests for independence and proportion tests. Analyses of TEM-1 and the combination of OXA-1/TEM-1 by individual institution also showed no significant associations (**Table 3**).

**Table 3:** Relative risk analysis of non-susceptibility to piperacillin/tazobactam in the presence of OXA-1, TEM-1, or OXA-1 and TEM-1 for all *E. coli* isolates.

All Isolates				
Gene	**RR of MIC >8 mg/m:**	**95% lower CI**	**95% Upper CI**	**P**
**OXA-1**	3.85	1.7	8.57	**<0.001**
**TEM-1**	0.92	0.39	2.16	0.424
**OXA-1 + TEM-1**	2.59	0.68	9.86	0.082
				
TAMC				
Gene	**RR of MIC >8 mg/m:**	**95% lower CI**	**95% Upper CI**	**P**
**OXA-1**	10.13	2.43	42.14	**<0.001**
**TEM-1**	2.05	0.39	10.61	0.2
**OXA-1 + TEM-1**	17.6	7.51	41.23	
				
MAMC				
Gene	**RR of MIC >8 mg/m:**	**95% lower CI**	**95% Upper CI**	**P**
**OXA-1**	22.94	2.96	177.96	**<0.001**
**TEM-1**	0.193	0.024	1.53	0.06
**OXA-1 + TEM-1**	1.54	0.211	11.25	0.33
				
BAMC				
Gene	**RR of MIC >8 mg/m:**	**95% lower CI**	**95% Upper CI**	**P**
**OXA-1**	0.43	0.055	3.36	0.21
**TEM-1**	1.39	0.35	5.55	0.32
**OXA-1 + TEM-1**	0			

Statistically significant values shown in bold.

We then investigated the association between the presence of the OXA-1 and/or TEM-1 genes and susceptibility to piperacillin/tazobactam in *K. pneumoniae* isolates, aggregating data from the three study sites due to the limited number of isolates available for individual site-specific analyses. MIC data were available for 83.6% (56/67) of the isolates, of which 58.9% (33/56) were found to be susceptible to piperacillin/tazobactam. Of the OXA-1-negative isolates, 93.9% (31/33) were susceptible to piperacillin/tazobactam, in contrast to only 8.7% (2/23) of OXA-1-positive isolates. Similarly, 84.0% (21/25) of TEM-1-negative isolates were susceptible, compared to 38.7% (12/31) of TEM-1-positive isolates. For isolates positive for both OXA-1 and TEM-1, susceptibility was observed in only 5.3% (1/19), compared to 95.2% (20/21) of isolates negative for both genes. Relative risk analysis of non-susceptibility to piperacillin/tazobactam demonstrated highly significant associations with OXA-1 (*P* < 0.001), TEM-1 (*P* = 0.003), and the combination of OXA-1 and TEM-1 (*P* < 0.001) in positive isolates.

**Figure 2:**
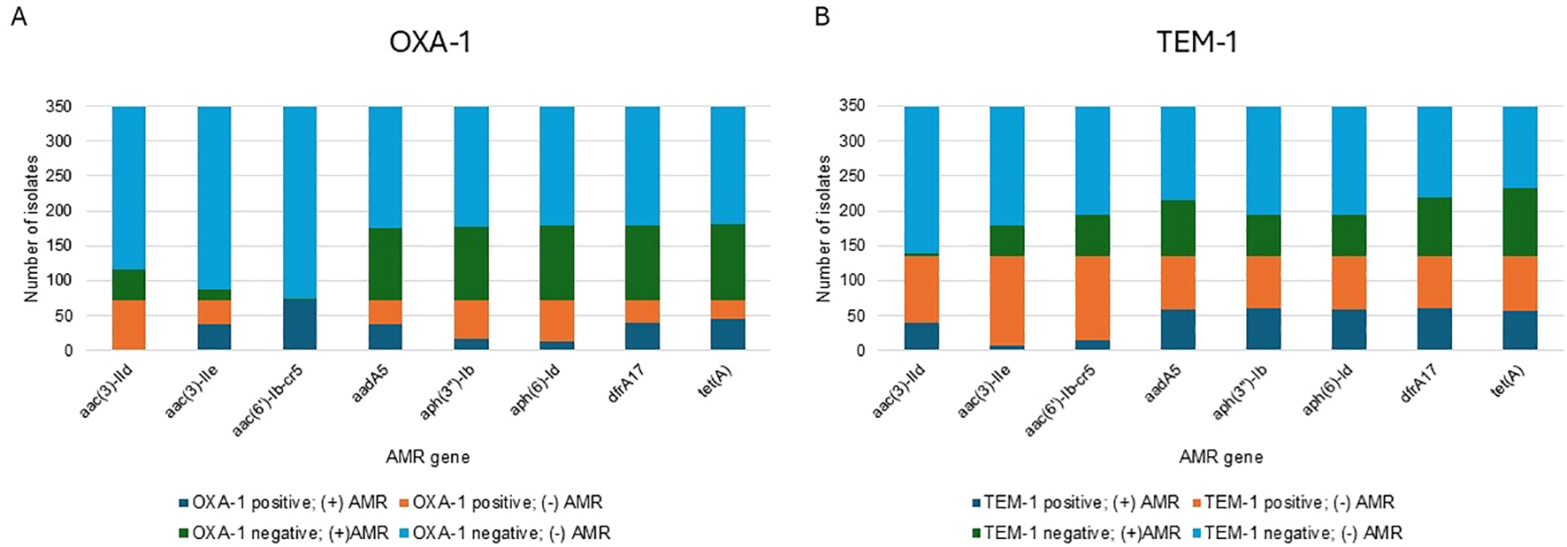
Distribution of antimicrobial resistance (AMR) gene combinations in *E. coli* isolates. **(A)** Relationship between OXA-1 and co-occurring AMR genes. Notably, the presence of aac(6')-Ib-cr5 always coincides with OXA-1, and this AMR gene is absent when OXA-1 is absent. **(B)** Relationship between TEM-1 and co-occurring AMR genes. The AMR gene aac(3)-IIe is rarely detected when TEM-1 is present but is more common when TEM-1 is absent.

We explored the association between the carriage of OXA-1 and/or TEM-1 genes and the presence of other resistance genes, focusing on those associated with resistance to aminoglycosides, fluoroquinolones, trimethoprim/sulfamethoxazole, and tetracycline. Specifically, we examined genes conferring resistance to the following drug classes: aac(3)-IId (aminoglycosides), aac(3)-IIe (aminoglycosides), aac(6’)-Ib-cr5 (aminoglycosides and fluoroquinolones), aadA5 (aminoglycosides), aph(3’’)-Ib (aminoglycosides), aph(6)-Id (aminoglycosides), dfrA17 (trimethoprim), and tet(A) (tetracycline). Interestingly, the aac(3)-IId gene was absent in OXA-1-positive variants (**Figure 2A**) and aac(3)-IIe was rarely found in TEM-1 positive variants (**Figure 2B**). Additionally, 98.6% (72/73) of OXA-1-positive variants carried the aac(6’)-Ib-cr5 gene, compared to only 0.72% (2/276) of OXA-1-negative variants. When we analyzed the relative likelihood of OXA-1 being present in relation to other resistance genes, OXA-1-positive variants showed a significant positive association with most of the tested resistance genes; however aph(6)-ld and aph(3”)-lb were significantly less likely to be present when OXA-1 was present. TEM-1-positive variants were significantly associated with the presence of aac(3)-lld, aph(3”)-lb, and aph(6)-ld and the absence of aac(6’)-lb-cr5 and aac(3)-lle (**Table 4**).

**Table 4:** Data showing relative likelihood of OXA-1 or TEM-1 being present in relation to the presence of other determinant of resistance genes.

All *E. coli* isolates. Risk of presence with OXA-1				
Resistance gene	RR	95% lower CI	95% upper CI	*P*
**aac(3)-lle**	9.33	5.43	16.03	**<0.001**
**aac(6’)-lb-cr5**	136.11	34.2	541.65	**<0.001**
**aadA5**	1.37	1.04	1.8	**0.011862**
**aph(3”)-lb**	0.58	0.37	0.92	**<0.001**
**aph(6)-ld**	0.46	0.28	0.78	**0.001722**
**dfrA17**	1.38	1.06	1.79	**0.007917**
**tet(A)**	1.58	1.25	1.99	**<0.001**
				

All *E. coli* isolates. Risk of presence with TEM-1				
Resistance gene	RR	95% lower CI	95% upper CI	*P*
**aac(3)-lld**	15.85	5.8	43.31	**<0.001**
**aac(3)-lle**	0.25	0.11	0.53	**0.000172**
**aac(6’)-lb-cr5**	0.4	0.24	0.68	**<0.001**
**aadA5**	1.17	0.91	1.51	0.117949
**aph(3”)-lb**	1.64	1.23	2.18	**<0.001**
**aph(6)-ld**	1.56	1.17	2.08	**0.001244**
**dfrA17**	1.14	0.89	1.46	0.154633
**tet(A)**	0.92	0.71	1.17	0.242268

Statistically significant values shown in bold.

Lastly, we assessed the association between gentamicin susceptibility and the presence of specific resistance in OXA-1- or TEM-1 variants. Gentamicin susceptibility data were available for 97.4% (340/349) of the *E. coli* isolates. For the OXA-1 gene no significant association with susceptibility to gentamicin was found. In contrast, the analysis for the TEM-1 gene revealed a significant association with gentamicin susceptibility (**Table 5A**). We evaluated the association of aac(3)-IIe, aac(6’)-Ib-cr5, aph(3’’)-Ib, and aph(6)-Id genes with gentamicin susceptibility in OXA-1 variants. A significant association between the presence of the aac(3)-IIe gene and susceptibility to gentamicin was found (**Table 5B**). We evaluated the association of aac(3)-IId, aac(3)-IIe, aac(6’)-Ib-cr5, and aph(6)-Id genes with gentamicin susceptibility in TEM-1 variants and found a significant association for the presence of the aac(3)-IId, aac(6’)-Ib-cr5, and aph(6)-Id genes (**Table 5C**).

**Table 5A:** Data showing association of gentamicin susceptibility and the presence of OXA-1 or TEM variants.

Gene	Status	TI	S	R	χ^2^	*P*	FET OR	*P*	PTS	*P*
OXA-1	P	72	49	23	1.573	0.210	0.697	0.211	-1.323	0.186
OXA-1	N	268	202	66						
TEM-1	P	133	89	44	5.392	**0.020**	0.569	**0.021**	-2.176	**0.030**
TEM-1	N	207	162	45						

P, positive; N, negative; TI, Total Isolates; S, susceptible; R, resistant; FET OR, Fisher's Exact Test (FET) Odds Ratio; PTS, Proportion Test Statistic. Statistically significant values shown in bold.

**Table 5B:** Data showing association of gentamicin susceptibility and the presence of the different genes in OXA-1 variant *E. coli* isolates.

Gene	Status	TI	S	R	χ^2^	*P*	FET OR	*P*
aac(3)-IIe	P	36	18	18	10.797	**0.001**	0.161	**0.002**
aac(3)-IIe	N	36	31	5				
aac(6')-Ib-cr5	P	71	49	22	2.161	0.142	6.600	0.254
aac(6')-Ib-cr5	N	1	0	1				
aph(6)-Id	P	13	10	3	0.574	0.449	1.709	0.452
aph(6)-Id	N	59	39	20				
aph(3”)-lb	P	16	13	3	1.647	0.199	2.407	0.209

P, positive; N, negative; TI, - Total Isolates; S, susceptible; R, resistant; FET OR, Fisher's Exact Test (FET) Odds Ratio; PTS, Proportion Test Statistic. Statistically significant values shown in bold.

**Table 5C:** Data showing association of gentamicin susceptibility and the presence of the different genes in TEM-1 variant *E. coli* isolates.

Gene	Status	TI	S	R	Chi-square	p-value	FET OR	p-value
aac(3)-IId	P	39	8	31	53.675	**<0.001**	0.041	**<0.001**
aac(3)-IId	N	94	81	13				
aac(3)-IIe	P	7	3	4	1.932	0.165	0.349	0.181
aac(3)-IIe	N	126	86	40				
aac(6’)-Ib-cr5	P	15	14	1	5.329	**0.021**	8.027	**0.048**
aac(6’)-Ib-cr5	N	118	75	43				
aph(6)-Id	P	59	27	32	21.436	**<0.001**	0.163	**<0.001**
aph(6)-Id	N	74	62	12				

P, positive; N, negative; TI, Total Isolates; S, susceptible; R, resistant; FET OR, Fisher’s Exact Test (FET) Odds Ratio; PTS, Proportion Test Statistic. Statistically significant values shown in bold.

## Discussion

This multi-center retrospective study investigated the prevalence of OXA-1 and TEM-1 genes in ESBL-producing *E. coli* and *K. pneumoniae* and their association with susceptibility to piperacillin/tazobactam. The study was conducted across three geographically distinct institutions: TAMC in Hawaii, MAMC in Washington, and BAMC in Southern Texas. This geographic diversity is crucial as it provides insights into regional variations in the prevalence of resistance genes and antibiotic susceptibility patterns. Our findings revealed significant differences in the distribution of resistance genes among the three institutions. For instance, the prevalence of the CTX-M-15 variant was highest at BAMC and lowest at TAMC, while the CTX-M-27 variant was most prevalent at TAMC. These variations underscore the importance of local epidemiological data in guiding empirical antibiotic therapy. The significant association between the OXA-1 gene and reduced susceptibility to piperacillin/tazobactam, particularly at TAMC and MAMC, highlights the need for region-specific treatment strategies (**Supplementary Figure 1**). In contrast, no significant association was observed between the TEM-1 gene and susceptibility to piperacillin/tazobactam. Additionally, we found that the presence of the aac(3)-IIe gene was significantly associated with gentamicin resistance in OXA-1 positive variants, while the aac(3)-IId, aac(6’)-Ib-cr5, and aph(6)-Id genes were significantly associated with gentamicin resistance in TEM-1 positive variants. This study emphasizes the necessity of comprehensive genetic profiling and local epidemiological surveillance to inform antibiotic stewardship programs and improve treatment outcomes for patients with ESBL-producing infections. By understanding the regional differences in resistance mechanisms, healthcare providers can tailor antibiotic therapies more effectively, reducing the risk of therapeutic failure and the spread of resistant pathogens.

The emergence of ESBL-producing Gram-negative pathogens has posed significant challenges in clinical settings, particularly in the treatment of infections caused by *E. coli* and *K. pneumoniae* (Mills et al., 2022; Kamau et al., 2023). Our study highlights the importance of understanding the genetic factors contributing to antibiotic resistance, as these can significantly impact treatment outcomes. The significant association between the OXA-1 gene and reduced susceptibility to piperacillin/tazobactam underscores the need for careful consideration of this gene when selecting treatment options for ESBL-producing infections.

The findings from our study are consistent with previous research that has demonstrated the role of OXA-1 in conferring resistance to penicillin/inhibitor combinations. Livermore et al. (2019) reported that the presence of OXA-1 was strongly associated with reduced susceptibility to piperacillin/tazobactam and amoxicillin/clavulanate in ESBL-producing *E. coli* isolates. Similarly, Walkty et al. (2022) found that OXA-1 was associated with elevated piperacillin/tazobactam MIC values among ESBL-producing *E. coli* clinical isolates. These studies, along with our findings, highlight the critical role of OXA-1 in resistance mechanisms and the potential for therapeutic failure when using penicillin/inhibitor combinations in the presence of this gene.

In contrast, our study did not find a significant association between the TEM-1 gene and susceptibility to piperacillin/tazobactam. This is in line with previous studies that have shown variable results regarding the impact of TEM-1 on resistance to penicillin/inhibitor combinations. For instance, Rodríguez-Villodres et al. (2018) demonstrated that piperacillin/tazobactam was less effective against ESBL *E. coli* carrying blaTEM due to increased copy numbers and transcription levels of the gene. However, other studies have not found a significant impact of TEM-1 on resistance, suggesting that additional factors may influence the efficacy of penicillin/inhibitor combinations in the presence of TEM-1.

Our study also explored the association between the presence of additional resistance genes and susceptibility to gentamicin. The significant association between the aac(3)-IIe gene and gentamicin resistance in OXA-1 positive variants, as well as the association between the aac(3)-IId, aac(6’)-Ib-cr5, and aph(6)-Id genes and gentamicin resistance in TEM-1 positive variants, underscores the complexity of resistance mechanisms in ESBL-producing pathogens. These findings suggest that the presence of specific aminoglycoside resistance genes can further complicate treatment outcomes and highlight the need for comprehensive genetic profiling of clinical isolates to guide antibiotic therapy.

The multi-center nature of our study provides valuable insights into the prevalence and distribution of resistance genes across different geographic regions and healthcare settings. The significant differences in the distribution of CTX-M-15, CTX-M-27, and CTX-M-14 variants among the three institutions underscore the importance of local epidemiological data in guiding empirical treatment decisions. The higher prevalence of CTX-M-15 in BAMC, for example, suggests that clinicians in this region may need to be particularly vigilant when selecting antibiotics for ESBL-producing infections.

Our study has several limitations that should be considered when interpreting the findings. The retrospective design may introduce selection bias, and the reliance on MIC data from routine clinical testing may not capture the full spectrum of resistance mechanisms. Another limitation of retrospective clinical data is the lack of MIC data for some isolates, specifically 13 ESBL OXA-1 positive isolates from BAMC were missing piperacillin/tazobactam MICs which could have affected the calculations for OXA-1 significance at that location. Additionally, the relatively small sample size for some gene variants may limit the generalizability of the findings. Future studies should aim to include larger sample sizes, prospective data collection, and gene copy number analysis to validate and expand upon our results.

In conclusion, our study highlights the critical role of the OXA-1 gene in conferring resistance to piperacillin/tazobactam in ESBL-producing *E. coli* and *K. pneumoniae*. The significant association between specific aminoglycoside resistance genes and gentamicin resistance further underscores the complexity of resistance mechanisms in these pathogens. Our findings emphasize the need for comprehensive genetic profiling and local epidemiological data to guide antibiotic therapy and improve treatment outcomes for patients with ESBL-producing infections.

## Materials and methods

### Bacterial isolates

A total of 416 ESBL-producing bacterial isolates from urine or blood specimens submitted to the clinical laboratories for testing in the three MTFs within the DHA as routine patient standard-of-care (SOC) submitted between January 2022 through December 2023 were retrospectively analyzed in this study. The three MTFs are Tripler Army Medical Center (TAMC) in Honolulu, Hawaii; Madigan Army Medical Center (MAMC) located on Joint Base Lewis-McChord, Washington; and Brooke Army Medical Center, located on Joint Base San Antonio-Fort Sam Houston, Texas. These institutions serve active duty service members, their families, military retirees and their families, veterans, and residents of the areas where they’re located, with TAMC serving the Indo-Pacific region, MAMC the Northwestern US region, and BAMC the South Central region of the US.

### Antimicrobial susceptibility testing

Bacterial identification and AST were performed as part of SOC from each patient using VITEK MS and Vitek 2 (bioMérieux, NC, USA) systems. The minimal inhibitory concentration (MIC) were interpreted per Clinical and Laboratory Standards Institute (CLSI) guidelines (CLSI, 2024). ESBL-producing organisms were determined based on Vitek 2 AST algorithm, which included non-susceptibility to ceftriaxone.

### Whole genome sequencing and data analysis

All ESBL-producing isolates from these and other MTFs are routinely submitted to the Multidrug-resistant organism Repository and Surveillance Network (MRSN) at Walter Reed Army Institute of Research (WRAIR), Silver Spring, MD, for whole genome sequencing (WGS) and they provided information about strain type and the presence of any antimicrobial resistance markers.

### Statistical analysis

Statistical analyses were performed to assess the association between the presence of resistance genes and susceptibility to piperacillin/tazobactam and gentamicin. The chi-square test for independence was used to determine whether there was a significant association between the presence of specific resistance genes and susceptibility to antibiotics. Fisher’s Exact Test was used as an alternative to the chi-square test for small sample sizes or when the assumptions of the chi-square test were not met. The proportion test was used to compare the proportions of susceptible and resistant isolates between different groups. The test statistic and p-values were calculated to determine if there were significant differences in the proportions. Relative risk was calculated to determine the probability of resistance to piperacillin/tazobactam given the presence of certain genes. Relative risk was also calculated for the probability of co-occurrence of antimicrobial resistance markers. All statistical analyses calculations were performed assessing significance at a *P* value equal to 0.05.

### Data presentation

The results of the statistical analyses were summarized in tables, including the total number of isolates, the number of susceptible and resistant isolates, chi-square test statistics, p-values, Fisher’s Exact Test odds ratios, and p-values for each gene and antibiotic combination.

### Software

All statistical analyses were performed using R software (version 4.0.3) and the stats and exact2x2 packages for chi-square and Fisher’s Exact Tests, respectively. The prop.test function was used for the proportion test.

## Data Availability

The original contributions presented in the study are included in the article/**Supplementary Material**. Further inquiries can be directed to the corresponding author.

## Supplementary material

Supplementary Table 1 All ESBL isolates examined in this study. 0=Not present, 1= Present, Pip/tazo= Measured MIC of piperacillin/tazobactam (µg/mL).

Supplementary Figure 1 Breakdown of piperacillin/tazobactam MICs by location and presence of AMR gene. The percentage of isolates for each AMR/location combination was calculated with different colors representing the MIC (ug/mL).
