# Supplementary figures and images for: Correlation of OXA-1 and TEM-1 genes with antibiotic resistance to piperacillin/tazobactam in ESBL-producing Enterobacterales: insights from a multi-center analysis
Source: Front Cell Infect Microbiol. 2026 Jan 12;15:1694724. doi: 10.3389/fcimb.2025.1694724 (PMC12832842; doi:10.3389/fcimb.2025.1694724)

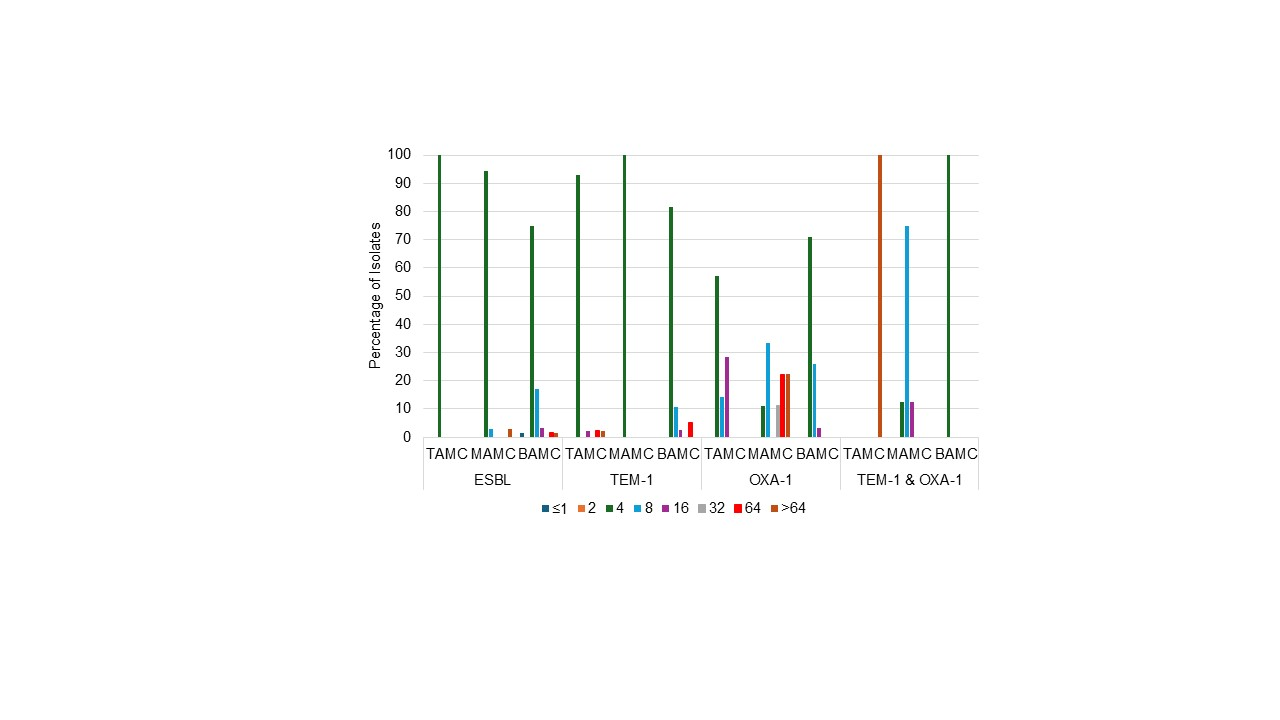

Supplement: Supplementary Figure 1 — Breakdown of piperacillin/tazobactam MICs by location and presence of AMR gene. The percentage of isolates for each AMR/location combination was calculated with different colors representing the MIC (ug/mL). [file Image1.tiff]
